# Genes regulated by DNA methylation are involved in distinct phenotypes during melanoma progression and are prognostic factors for patients

**DOI:** 10.1002/1878-0261.13185

**Published:** 2022-02-04

**Authors:** Debora D’Angelo Papaiz, Flávia Eichemberger Rius, Ana Luísa Pedroso Ayub, Clarice S. Origassa, Hemant Gujar, Diogo de Oliveira Pessoa, Eduardo Moraes Reis, Jérémie Nsengimana, Julia Newton‐Bishop, Christopher E. Mason, Daniel J. Weisenberger, Gangning Liang, Miriam Galvonas Jasiulionis

**Affiliations:** ^1^ Pharmacology Department Escola Paulista de Medicina Universidade Federal de São Paulo Brazil; ^2^ 5116 Department of Urology University of Southern California Los Angeles CA USA; ^3^ Instituto de Química Universidade de São Paulo Brazil; ^4^ Biostatistics Research Group Faculty of Medical Sciences Population Health Sciences Institute Newcastle University UK; ^5^ University of Leeds School of Medicine UK; ^6^ Department of Physiology and Biophysics Weill Cornell Medicine New York NY USA; ^7^ 5116 Department of Biochemistry and Molecular Medicine University of Southern California Los Angeles CA USA

**Keywords:** DNA methylation, epigenetics, gene body, melanoma, prognosis, gene promoter

## Abstract

In addition to mutations, epigenetic alterations are important contributors to malignant transformation and tumor progression. The aim of this work was to identify epigenetic events in which promoter or gene body DNA methylation induces gene expression changes that drive melanocyte malignant transformation and metastasis. We previously developed a linear mouse model of melanoma progression consisting of spontaneously immortalized melanocytes, premalignant melanocytes, a nonmetastatic tumorigenic, and a metastatic cell line. Here, through the integrative analysis of methylome and transcriptome data, we identified the relationship between promoter and/or gene body DNA methylation alterations and gene expression in early, intermediate, and late stages of melanoma progression. We identified adenylate cyclase type 3 (*Adcy3*) and inositol polyphosphate 4‐phosphatase type II (*Inpp4b*), which affect tumor growth and metastatic potential, respectively. Importantly, the gene expression and DNA methylation profiles found in this murine model of melanoma progression were correlated with available clinical data from large population‐based primary melanoma cohorts, revealing potential prognostic markers.

## Graphical abstract

Epigenetic alterations related to aberrant gene expression are important regulators of malignant transformation and melanoma progression. Here, we integrated transcriptome and methylome data from a linear model of murine melanoma progression and identified promoter and/or gene body DNA methylation profiles that induced gene expression changes related to melanocyte transformation and metastasis. We defined *Adcy3* and *Inpp4b* as key regulators of metastasis *in vivo* and observed a correlation of the identified profiles/genes to patient survival, thus demonstrating their potential use as prognostic markers.

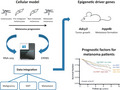

**Abbreviations**
5azaCdR: 5‐Aza‐2′‐deoxycytidine
5mC: 5‐methylcytosine
Actb: beta‐actin
ADCY3: adenylate cyclase type 3
AKT: protein kinase B
ANOVA: analysis of variance
bp: base pairs
cDNA: complementary DNA
CpG: cytosine‐phosphate‐guanine
CREB: cAMP‐responsive element binding protein
Dct: dopachrome tautomerase
EMT: epithelial‐to‐mesenchymal transition
EPAC: exchange factor directly activated by cAMP
ERK: extracellular signal‐regulated kinases
ERRBS: enhanced reduced representation bisulfite sequencing
FBS: fetal bovine serum
HDAC: histone deacetylase
INPP4B: inositol polyphosphate 4‐phosphatase type II
KEGG: Kyoto encyclopedia of genes and genomes
logFC: logarithm fold change
LRRK2: leucine‐rich repeat kinase 2
MAPK: mitogen‐activated protein kinase
MITF: melanocyte‐inducing transcription factor
Mlana: melanoma antigen recognized by T cells 1
MMP: matrix metalloproteinase
MSS: melanoma‐specific patient survival
PKA: protein kinase A
PMA: phorbol *12‐*myristate *13‐*acetate
PPI: protein–protein interactions
RNA‐seq: ribonucleic acid sequencing
RPMI: Roswell park memorial institute
RT‐qPCR: real‐time quantitative polymerase chain reaction
SEM: standard error of mean
SGK3: serum/glucocorticoid regulated kinase family member 3
siRNA: small interfering RNA
SKCM: skin cancer melanoma
TCGA: the cancer genome atlas
TP53: transformation‐related protein 53
TSA: trichostatin A
TSS: transcriptional start site
Tyrp1: tyrosinase‐related protein 1
UV: ultraviolet

## 1 Introduction

Epigenetic changes in regulatory regions of the genome are involved in tumorigenesis and can drive cancer development and progression by altering gene expression. Human cancers display global DNA hypomethylation concomitant with specific promoter DNA hypermethylation correlating with oncogene activation and gene silencing of tumor suppressor genes, respectively [1]. While promoter DNA methylation is related to gene silencing, gene body DNA methylation is a feature of actively transcribed genes [2] and is also a prominent target for cancer treatment, as treatment with DNA demethylating agents can lead to downregulation of over‐expressed genes, such as oncogenes and those involved in MYC‐regulated metabolic pathways [3]. Cancer‐associated DNA methylation aberrancies are not only therapeutic targets but also effective biomarkers for early detection, prognosis, and risk stratification of cancer patients [4, 5].

Altered epigenetic mechanisms are hallmarks of human cutaneous melanoma, a highly aggressive and metastatic cancer [6, 7]. As DNA methylation plays an important role in melanoma initiation and progression, several DNA methylation biomarkers have been identified and showed association with overall patient survival, drug resistance as well as drug sensitivity [8]. However, the exact mechanism of how DNA methylation is directly involved in gene regulation and how epigenetically regulated genes influence melanoma initiation and progression are still not clear. Mouse models of melanoma have been developed and reported [9], but these usually fail to address tumor complexity and cellular heterogeneity.

In this study, we used a mouse model consisting of four cell lines that represent melanoma tumor progression from melanocytes (melan‐a), to premalignant melanocytes (4C), to nonmetastatic melanoma cells (4C11‐), and finally to metastatic melanoma cells (4C11+) to understand how DNA methylation aberrancies alter gene expression and drive melanoma development and progression. This model was established by our group through continuous stress of murine melanocytes, melan‐a, a spontaneously immortalized cell line that retains all characteristics of melanocytes except senescence, and are nontumorigenic [10]. Melan‐a melanocytes were subjected to sequential cycles of deadhesion for 96 h followed by adhesion. After the fourth cycle, it gave origin to the cell line 4C, a premalignant, undifferentiated/mesenchymal, and nontumorigenic cell line. After limiting dilution of 4C spheroids formed by another deadhesion, it was established the cell line 4C11−, an undifferentiated/mesenchymal and slow‐growth melanoma cell line capable of developing tumors *in vivo*. After the spontaneous loss of p53, 4C11− gave origin to the differentiated/pigmented and highly proliferative 4C11+ cell line, capable of forming fast‐growing tumors and developing lung metastasis *in vivo* [11, 12, 13, 14]. Alterations in the global level of 5mC content and histone modifications, as well as in the expression of several epigenetic machinery components were previously described in this model [14, 15, 16, 17, 18]. Moreover, genes presenting altered expression along melanoma progression as a result of aberrant DNA methylation in its promoters were also identified, as well genes presenting promoter DNA methylation status and/or gene expression with prognostic value.

Here, we integrated transcriptome (RNA‐seq) and methylome (ERRBS) data for all four cell lines to gain insight into the promoter and gene body DNA methylation driver events that influence gene expression during melanoma progression. Furthermore, we investigated the transcription and DNA methylation signatures derived from this model and validated our findings in independent cohorts of primary melanomas (Leeds melanoma transcription cohort) [19], primary and metastatic cutaneous melanomas (The Cancer Genome Atlas, TCGA, https://www.cancer.gov/tcga; and the Sweden DNA methylation cohort, http://shiny.maths.usyd.edu.au/melanomaExplorer/), for their prognostic relevance.

## 2 Materials and methods

### 2.1 Cell culture

Melan‐a [10] and their derived cell lines 4C, 4C11−, and 4C11+ [12, 14] were cultured in RPMI 1640 medium pH6.9 supplemented with 5% FBS and 1% penicillin (100 U·mL^−1^) and streptomycin (100 µg·mL^−1^) at 37 °C in 5% CO_2_ humidified atmosphere. The melan‐a cell line also had its medium supplemented with 200 nm of PMA (Phorbol 12‐myristate‐13‐acetate).

### 2.2 RNA sequencing (RNA‐seq)

Total RNA was isolated from each murine cell line in triplicate using TRIzol reagent following the manufacturer’s protocol (Thermo Fisher Scientific Inc., Carlsbad, CA, USA). After extraction, libraries were prepared using the Illumina TruSeq™ Stranded Total RNA Library Prep Kit with Ribo‐Zero Gold (cat. # RS‐122‐2001, Illumina Inc., San Diego, CA, USA). Libraries were quantified and then sequenced using the Illumina HiSeq 2500 system (SBS Kit V4 250 cycle kit, Illumina Inc., San Diego, CA, USA). Differential expression analyses between pairs of cell lines were performed after data normalization using the *voom* function of the ‘limma’ package [20] in r computing language. A significance threshold of log_2_ ratios ≥ |2| and Benjamini–Hochberg adjusted *P* ≤ 0.01 was used.

### 2.3 Enhanced reduced representation bisulfite sequencing (ERRBS)

We performed ERRBS [21] to identify base pair‐level DNA methylation profiles for each cell line with special attention to CpG islands and shores. Genomic DNA from each cell line of the murine model was extracted in triplicate using Gentra Puregene Cell Kit (Qiagen, Hilden, Germany) as recommended by the manufacturer. ERRBS libraries were constructed and then sequenced using the Illumina HiSeq 2500 system at the Epigenomics Core Laboratory of Weill Cornell Medical College (New York, USA).

Pairwise comparisons of differential DNA methylation analyses were conducted between mouse cell lines using the r package *methylkit* [22]. *P*‐values were corrected to *q*‐values using the package default method: SLIM and CpGs [23] were considered differently methylated if *q*‐value ≤ 0.01 and ≥ 25% DNA methylation difference between compared lineages. Promoter regions were defined as located ±500 bp from the transcription start site (TSS) and gene body regions were located between TSS +500 bp and the transcription end site (TxEnd). We used the *genomicranges* package [24] in R programming language to overlay differentially methylated CpGs with their gene annotation. A gene region was determined to be differentially methylated if it contained at least three CpGs within the region.

### 2.4 Transcriptome and methylome data integration

Differentially methylated genes with gene expression changes were selected in pairwise comparisons between cell lines when they presented: (a) a positive correlation between gene expression and gene body DNA methylation or (b) a negative correlation between gene expression and promoter DNA methylation. We defined a *malignancy transformation signature* as the collection of genes with significant alterations in DNA methylation and gene expression changes in 4C, 4C11‐, and 4C11+ cells compared to the melan‐a parental cell line. Similarly, a *metastasis signature* was defined as genes altered only in 4C11+ cells when comparison to melan‐a, 4C, and 4C11− cells. Finally, the *EMT signature* highlights genes altered in the ‘mesenchymal‐like’ 4C and 4C11− cell lines when compared to differentiated melan‐a and 4C11+ cells. Therefore, each signature is a list of genes significantly up‐ or downregulated by DNA hypo‐ or hyper‐methylation. Separated signatures were obtained from promoter and gene body DNA methylation.

### 2.5 Gene enrichment analysis

Genes selected in each signature were analyzed for pathway enrichment and biological process enrichment using KEGG and Gene Ontology, respectively. Both analyses were conducted using the R package *clusterProfiler* [25] and adjusted *P* ≤ 0.05 was used.

### 2.6 Quantitative real‐time PCR

We isolated total RNA in triplicate from all four cell lines TRIzol (Invitrogen) reagent and synthesized cDNA using QuantiTect Reverse Transcription Kit (Qiagen) according to the manufacturer’s protocol. RT‐PCR assays were performed using Fast Sybr Green Master Mix (Thermo Fisher Scientific Inc.) on an Applied Biosystems 7500 Real‐Time PCR system (Thermo Fisher Scientific Inc.). The specific RT‐PCR primer sequences used were as follows: *Adcy3* forward, 5′ AGG GCA TCG AAA CCT ACC TC 3′; *Adcy3* reverse, 5′ CAT TGG GCT CCT TGG TCT CG 3′; *Inpp4b* forward, 5′ CAC CGT GGA GAA TAG GTC CG 3′; *Inpp4b* reverse, 5′ GAC AGG AGC CAC AAG ATC CC 3′; *Lrrk2* forward, 5′ ACC CTG TAT CCC AAT GCT GC 3′; *Lrrk2* reverse, 5′ CAT TCC CCC TGG CAA CTT CA 3′; *β‐actin* forward, 5′ ACC GTG AAA AGA TGA CCC AG 3′; *β‐actin* reverse, 5′ GTA CGA CCA GAG GCA TAC AG 3′. Relative gene expression levels were quantified using $2^{‐\Delta \Delta C_{q}}$ and normalized to beta‐actin (*Actb*).

### 2.7 Epigenetic inhibitor treatments

To analyze epigenetic regulation of candidate gene expression, we treated each murine cell line with 1 µm 5‐Aza‐2′‐deoxycytidine (5‐aza‐CdR, Sigma‐Aldrich, San Luis, MO, USA) for 72 h, 40 nm Trichostatin A (TSA—Merck Millipore, Burlington, MA, USA) for 18 h, or combination treatment of 1 µm 5‐aza‐CdR for 72 h followed by 40 nm TSA for 18 h. Dosage and time used for treatments were based on previous laboratory assays taking into consideration cell viability and drug cytotoxicity [17]. All drug treatments were performed in biological triplicates. Control experiments were performed without the addition of either drug. After drug treatments, we extracted total RNA from each cell line, then synthesized cDNAs, and analyzed gene expression using RT‐qPCR as described previously. Relative gene expression was conducted in reference to untreated cells and normalized to *Actb* expression levels.

### 2.8 Patient cohorts and survival analyses

The Leeds Melanoma Cohort (accession number EGAS00001002922) contains gene expression profiles for 703 primary tumors (drug‐naïve) and was used to assess the prognostic value of DNA methylation signatures. Gene expression levels from each melanoma signature group (malignancy transformation, metastasis, and EMT) were averaged into one score after z‐transformation (mean 0 and variance 1). Upregulated and downregulated genes were considered separately and jointly with −1 weighting applied to downregulated genes in the combined scores. Next, signature scores were tested for their association with melanoma‐specific survival using Cox proportional hazard regression and Kaplan–Meier plots after dichotomization by the median. Scores were also tested for their associations with Breslow thickness and immune cell scores inferred from gene expression as previously reported [19]. These analyses were conducted in stata 14 (StataCorp, College Station, TX, USA).

We accessed publicly available human melanoma datasets including The Cancer Genome Atlas (TCGA SKCM) (gene expression and DNA methylation), the Sweden cohort (gene expression and DNA methylation), and the Hunter Australians cohort (mRNA) using the melanoma explorer tool (http://shiny.maths.usyd.edu.au/melanomaExplorer/) [26]. Using these data sets, we stratified tumor samples by their gene expression and DNA methylation levels with tumors displaying gene expression or DNA methylation values below the lower threshold (25%) and tumors displaying gene expression or DNA methylation values above the upper threshold (75%).

Patient survival over time for each group was visualized using Kaplan–Meier plots and the significance of survival profile differences was computed using the log‐rank test. Melanoma data sets from GSE3189, GSE8401, and GSE19234 studies were accessed using Oncomine [27]. TCGA melanoma protein expression data were accessed using the TRGAted web tool (https://github.com/ncborcherding/TRGAted) [28]. Information about each cohort, number of samples, methodologies, references, and tools is available in Table S1.

### 2.9 siRNA transfection

To identify potential role of selected genes, we transfected mouse cell lines with *Inpp4b* or *Adcy3* siRNAs designed by fabricant (IDT) using Lipofectamine (Invitrogen) for 24h. Scrambled nontarget siRNA sequences were used as negative controls.

### 2.10 *In vivo* tumor formation and metastasis assays

Female 6‐ to 8‐week‐old C57Bl/6 mice were obtained from Biotério Central, Universidade Federal de São Paulo, Brazil. Animals were maintained on a 12 h light/dark cycle and had free access to food according to the International Guiding Principles for Biomedical Research Involving Animals (Genebra, Geneva, Switzerland). The ethical committee Comissão de Ética no Uso de Animais (CEUA) of UNIFESP approved all animal experiments under the identification 8221090519. 4C11+ cells (2.10^5^) transfected with *Adcy3* or *Inpp4b* siRNAs were subcutaneously injected into mice. Tumor growth was measured with a caliper and after 15 days mice were euthanized. Tumors were removed and tumor masses were measured using an analytical scale. For metastasis assays, siRNA‐transfected 4C11+ cells (2.10^5^) were injected into the lateral tail vein and lungs were removed 21 days later for the visual analysis of metastatic foci.

### 2.11 Statistical analyses

Statistical analysis of cell line data was conducted using graphpad prism 8.0.2 software (GraphPad Software, San Diego, CA, USA). Two‐way ANOVA with multiple comparisons test was used when comparing three or more groups using Tukey test for correction. One‐way ANOVA was used for mean comparisons followed by the Tukey multiple comparison test.

## 3 Results

### 3.1 Gene expression alterations are differentially regulated by promoter or gene body DNA methylation during melanoma progression

To unravel how DNA methylation directly alters gene expression and drives the development and progression of melanoma, we used a mouse model [12] developed by applying continuous stress to spontaneously immortalized melan‐a cells [10]. Melan‐a cells also express genes responsible for the melanocytic pathway observed in normal melanocytes, such as *Mitf*, *Tyrp1*, *Mlana,* and *Dct*. This first involved subjecting melan‐a cells to four anchorage‐impediment cycles, giving origin to the 4C premalignant, nontumorigenic cell line. 4C spheroids were subjected to another deadhesion cycle, giving rise to the 4C11− cell line. 4C11− cells are slow‐growing melanoma cells capable of developing tumors *in vivo*. 4C11− cells exhibited spontaneous *Trp53* deletion in culture and became highly proliferative. These 4C11− *Trp53*‐deleted cells were subsequently termed 4C11+ cells and are also capable of developing lung metastasis *in vivo* [11, 12]. Thus, our melanoma model consists of four cell lines that show linear melanoma progression from melanocytes to premalignant melanocytes to nonmetastatic melanoma cells to metastatic melanoma cell types.

**Fig. 1:**
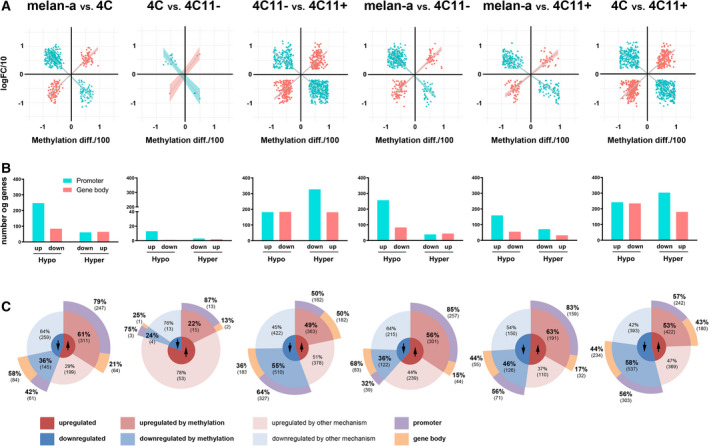
DNA methylation at gene body and promoter regions regulates gene expression in distinct stages of melanoma progression. (A) Scatterplots of genes regulated by promoter (blue) or gene body (pink) DNA methylation illustrating positive or negative correlations between gene expression (logFC/10) and DNA methylation (methylation difference/100) in each pairwise comparison between cell lines. (B) Bar plot with the number of genes differently expressed (up: upregulated, down: downregulated) regulated by DNA methylation (hyper: hypermethylated, hypo: hypomethylated) in promoter (blue) and gene body (pink) regions based on pairwise comparison between cell lines. (C) Pie charts showing percentages and number of differently expressed genes and promoter or gene body DNA methylation. Arrows indicate up‐ or downregulated gene expression. For methylation analysis, triplicates of each cell line were sequenced, and for RNA‐seq, the same was performed except for melan‐a cell line that was sequenced in duplicate. melan‐a: parental nontumorigenic melanocytes; 4C: premalignant undifferentiated melanocytes; 4C11−: nonmetastatic undifferentiated melanoma cells; 4C11+: metastatic differentiated melanoma cells.

As mentioned before, our melanoma progression cellular model was established through continuous stress instead of genetic manipulation, resulting in epigenetic alterations both very early and late in tumor progression as previously described [12, 13]. In a recent study, Preston‐Alp and colleagues showed that UV radiation alters DNA methylation both in primary human melanocytes and in murine melan‐a melanocytes in CpG sites found to be prognostic of overall survival of melanoma patients [29]. Specifically, changes in DNA methylation, a common epigenetic modification in human cancer genomes, were abundant in our cellular model. Studies have demonstrated a negative correlation between gene expression and promoter DNA methylation, but a positive correlation of gene expression with gene body DNA methylation [3, 30]. Therefore, we analyzed previous data from RNA‐seq (transcriptome) [13] and ERRBS (methylome) of the four cell lines and integrated these data to unravel how methylation in different regions of the gene may affect gene expression. Our approach was to select genes that showed, in each comparison expression, changes positively correlated with alterations in gene body DNA methylation, as well as genes that showed a negative correlation between changes in expression and promoter DNA methylation (Fig. 1A,B). We identified some genes that had both gene body and promoter methylation differences correlated with altered gene expression; however, most of them showed < 20% difference in methylation changes between cell lines. Genes that showed methylation in both regions and had a difference bigger than 20% between cell lines in methylation were not used in further analysis and can be found in Table S2. We first compared the data of melanocytes (melan‐a) with the cell lines representing stages of melanoma progression (4C, 4C11− and 4C11+). Interestingly, we observed larger numbers of genes upregulated by promoter DNA hypomethylation when comparing melan‐a cells to the other three cell types (*n* = 247 for melan‐a vs. 4C, *n* = 257 for melan‐a vs. 4C11‐, and *n* = 159 for melan‐a vs. 4C11+) than genes downregulated by promoter DNA hypermethylation after comparing melan‐a to 4C (*n* = 61), 4C11− (*n* = 39), or 4C11+ cells (*n* = 71) (Fig. 1C), suggesting that transformed melanoma cells display widespread promoter DNA hypomethylation, in agreement with findings in melanoma [31] and other human cancer types [32]. Differently, only slight changes were observed in the number of genes downregulated by gene body DNA hypomethylation after comparing melan‐a to 4C (*n* = 84), 4C11− (*n* = 83), or 4C11+ (*n* = 55) or genes upregulated by gene body DNA hypermethylation (*n* = 64 for melan‐a vs. 4C, *n* = 44 for melan‐a vs. 4C11−, and *n* = 32 for melan‐a vs. 4C11+), suggesting that changes in gene body methylation are less prevalent during initial melanoma progression. When comparing gene regulation between 4C and 4C11− cells, we identified a small number of genes with changes in promoter or gene body methylation, most likely due to their very similar phenotypic characteristics [13]. When comparing cells corresponding to initial stages of melanoma progression (4C and 4C11−) to late‐stage 4C11+ cells, we observed more pronounced gene expression changes that are associated with gene body or promoter DNA methylation aberrancies (Fig. 1A,B). Large numbers of genes downregulated by promoter DNA hypermethylation and gene body DNA hypomethylation were observed in 4C11+ cells compared to 4C (*n* = 303 and *n* = 234, respectively) and 4C11− (*n* = 327 and *n* = 183, respectively). The number of genes upregulated by promoter DNA hypomethylation and gene body DNA hypermethylation was also significant (respectively, *n* = 242 and *n* = 180 for 4C vs. 4C11+, and *n* = 182 and *n* = 182 for 4C11− vs. 4C11+).

We also observed the percentages of up‐ or downregulated genes that were correlated with gene body or promoter DNA methylation changes in the pairwise comparison between each cell line (Fig. 1C). We observed that 56–63% of all upregulated genes and 36–46% of all downregulated genes were correlated with DNA methylation changes when comparing melan‐a cells to the other three cell types. Most of the upregulated genes had their expression regulated by promoter DNA methylation changes, highlighting that promoter DNA hypomethylation is more prevalent than promoter DNA hypermethylation in regulating gene expression. In comparing 4C11+ cells to 4C and 4C11‐ cells, we identified slightly lower percentages of upregulated genes (49–53%) than downregulated genes (55–58%) that were correlated with DNA methylation changes. Interestingly, the contribution of gene body DNA methylation changes to the progression from early to late stages of melanoma progression was pronounced both for up‐ (43–50%) and downregulated genes (36–44%). 4C and 4C11‐ cells showed few gene expression differences, most of which were not due to DNA methylation alterations, suggesting that other mechanisms may be responsible for gene regulation changes during the progression from premalignant melanocytes (4C) to nonmetastatic melanoma cells (4C11−). Our findings suggest that DNA methylation is substantially involved in altering gene regulation along melanoma progression, highlighting epigenetic plasticity during tumorigenesis and tumor aggressiveness.

### 3.2 DNA methylation drives melanocyte malignant transformation, EMT, and metastasis in the murine melanoma model

Genetic and epigenetic aberrations have been described during tumorigenesis; however, the majority of the alterations defined to date are passenger events that do not contribute to functional gene expression changes. As a result, characterizing genetic and epigenetic drivers of disease initiation and progression remains elusive. To determine potential epigenetic drivers of melanoma, we performed unsupervised hierarchical clustering of differentially expressed genes regulated by promoter or gene body DNA methylation in our murine model. A heatmap representation of the clustered data using log fold change (logFC) of differential expression levels revealed distinct gene expression profile alterations (Fig. S1). Since the murine melanoma model comprises different stages of melanoma progression in a linear fashion, we decided to identify differentially expressed genes that were regulated by promoter or gene body DNA methylation during melanoma progression and metastasis by stratifying the data into three signatures: *Malignancy*, *Epithelial‐to‐mesenchymal transition* (EMT), and *Metastasis*. These signatures were chosen based on morphological phenotype [13, 17], functional characteristics, such as *anoikis* resistance [33], cell proliferation [11, 14], migration [14], invasion [14], MMPs activity [33], *in vivo* tumor growth and lung colony formation [11, 14], and expression of epithelial and mesenchymal markers (data not shown) and transcription factors that induce EMT [13]. The transcriptome analysis of melan‐a, 4C, 4C11−, and 4C11+ emphasized these changes between cell lines and showed the potential in discovering prognostic markers through malignant transformation, EMT, and metastasis signatures [13].

**Fig. 2:**
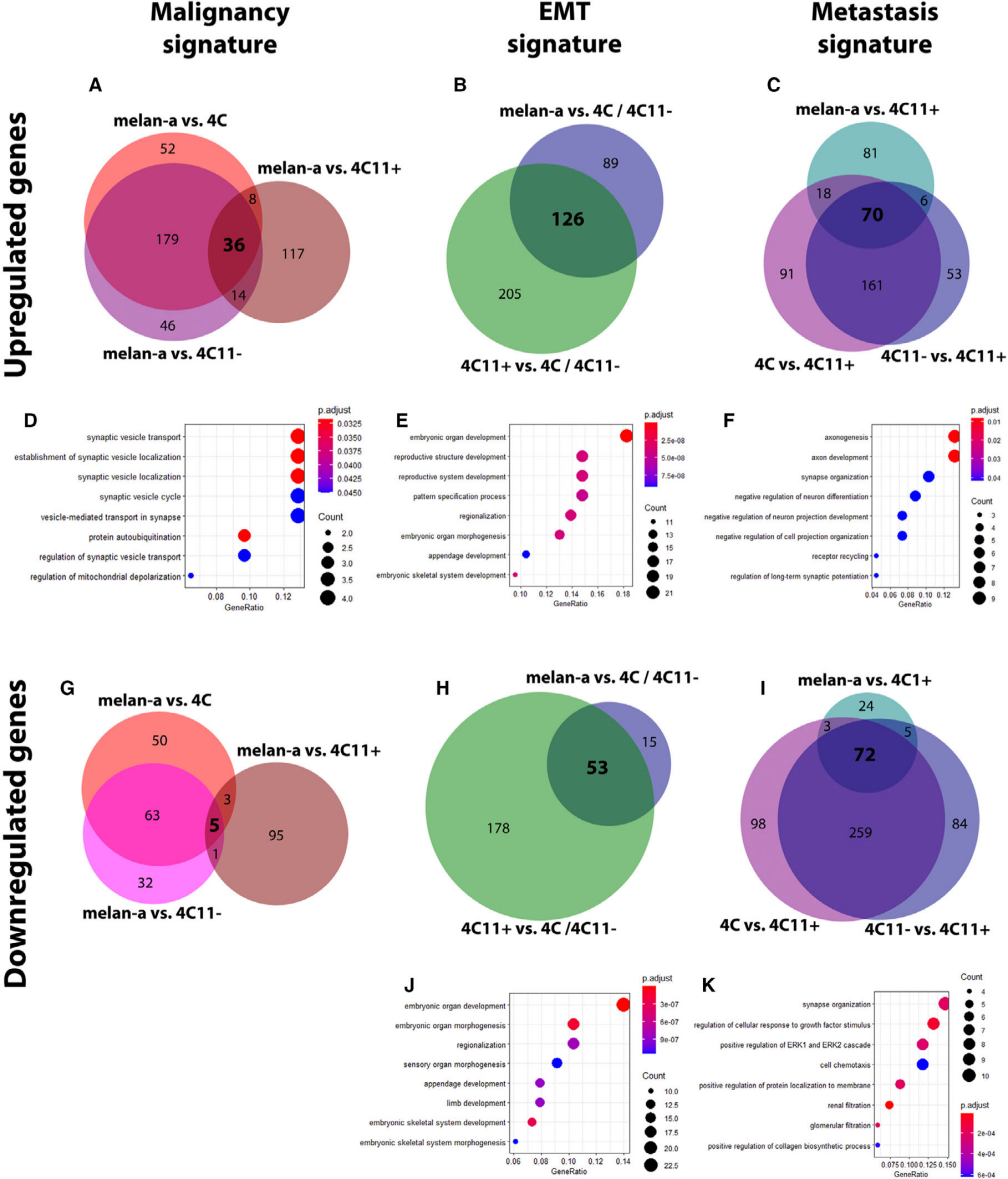
Identification of up‐ and downregulated genes regulated by DNA methylation in malignancy, EMT, and metastasis signatures provide insights about its role in melanoma progression. Venn diagrams of genes identified in each signature illustrating the number of genes potentially up‐ (A–C) or downregulated (G–I) by promoter DNA methylation according to signatures of malignancy (alterations in all cell lines compared to melan‐a melanocytes) (A and G), EMT (alterations in the 4C/4C11− mesenchymal‐like compared to melan‐a/4C11+ differentiated cells) (B and H), and metastasis (alterations only in the metastatic 4C11+ cells compared to the other three cell lines) (C and I). Below each signature, biological processes enriched among up‐ (D–F) and downregulated (J, K) genes of each intersection are shown. For methylation analysis, triplicates of each cell line were sequenced, and for RNA‐seq the same was performed except for melan‐a cell line that was sequenced in duplicates. melan‐a: parental nontumorigenic melanocytes; 4C: premalignant undifferentiated melanocytes; 4C11−: nonmetastatic undifferentiated melanoma cells; 4C11+: metastatic differentiated melanoma cells.

The malignancy signature genes (*n* = 41) were identified as those in common after Venn diagram analyses of 4C, 4C11−, and 4C11+ data compared to melan‐a cells (Fig. 2A). Similarly, the EMT signature genes (*n* = 179) were identified between cells presenting a differentiated phenotype (melan‐a and 4C11+) and those with an undifferentiated/mesenchymal phenotype (4C and 4C11−) (Fig. 2B). At last, we defined the metastasis signature as the set of genes (*n* = 142) that are differentially expressed in malignant 4C11+ cells as compared to melan‐a, 4C, and 4C11− cells (Fig. 2C). We also identified upregulated and downregulated genes in each signature due to either promoter or gene body DNA methylation, as shown in Table S3.

Next, we evaluated the representation of specific biological processes and molecular pathways among up‐ and downregulated genes in each signature (Fig. 2). Genes upregulated in the malignancy signature were enriched in pathways related to synaptic processes and neural tissues (Fig. 2D). The enrichment of neural‐related processes was also observed before by others, due to melanocytes and neural tissues are derived from neural crest cells [34]. Upregulated EMT signature genes were enriched for processes involved in regulating early stages of cell development (Fig. 2E), suggesting that these genes are important for an undifferentiated state. With regard to the metastasis signature, the upregulated genes were enriched for neural differentiation processes (Fig. 2F). We did not observe the enrichment of functional categories among downregulated genes in the malignancy signature, as only a small number of genes were identified. Downregulated genes present in the EMT signature were enriched for processes involving early stages of cell development (Fig. 2J), while the downregulated genes in metastasis signature were enriched for response to growth stimulus factors and positive regulation of the ERK1 and ERK2 signaling pathway cascade (Fig. 2K), a known disrupted pathway in human melanoma [35].

### 3.3 Gene signatures identified in the murine melanoma model correlate with Breslow thickness, tumor immune cell score, and patient survival in melanoma patients

**Fig. 3:**
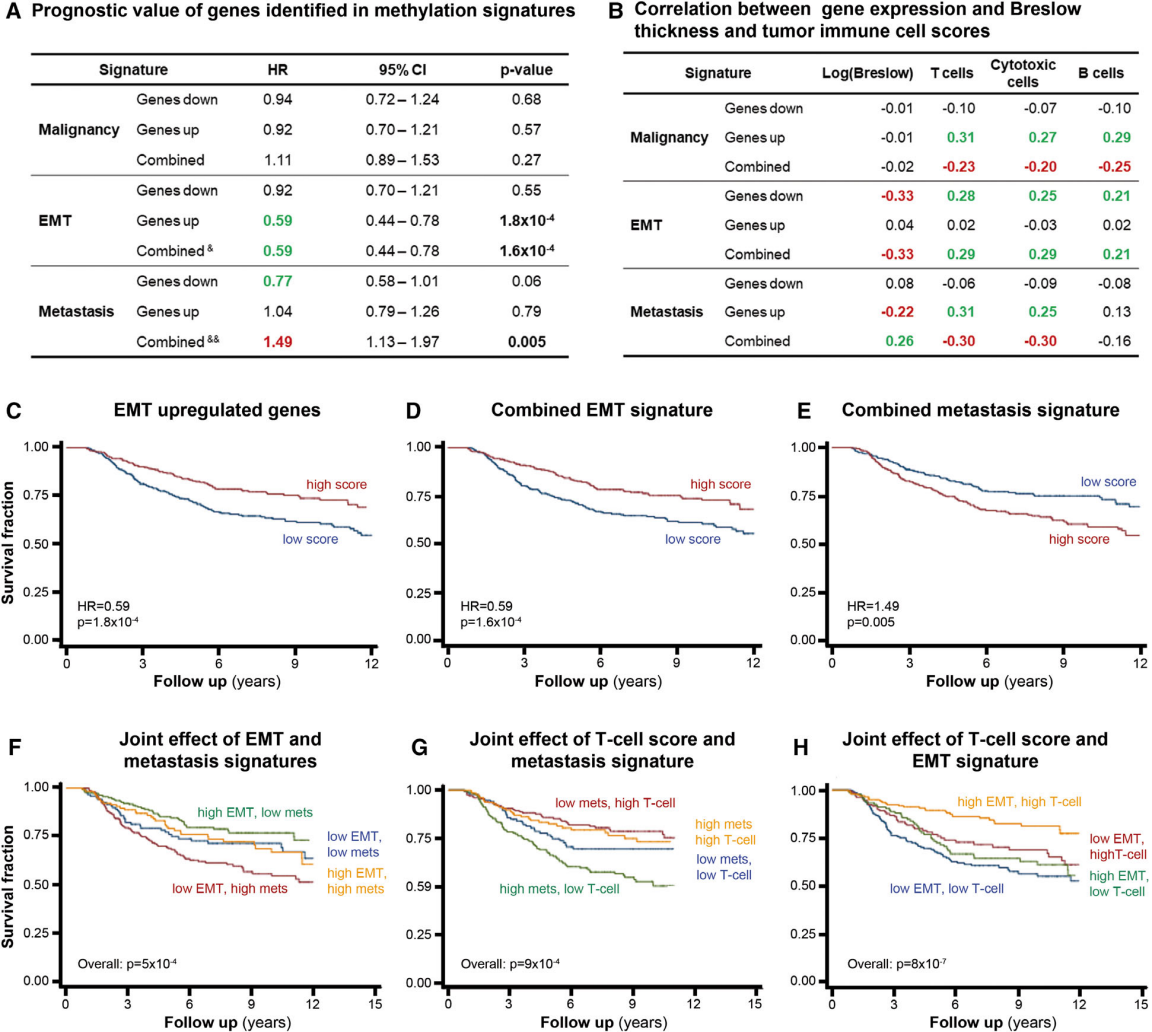
Gene signatures as prognostic factors for melanoma patients. (A, B) Upregulated, downregulated, and the combination of up‐ and downregulated genes identified in each signature (malignancy, EMT, metastasis), and the corresponding values of hazard ratio (HR), confidence interval (CI) and *P*‐value (A), as well as Breslow thickness, T‐cell score, cytotoxic cell score, and B‐cell score (B) in the Leeds Melanoma Cohort. In (A), green color indicates low risk, and red, high risk. In (B), green color indicates positive correlation, and red negative correlation. &: The metastasis signature effect is weaker when adjusting for T‐cell score (*P* = 0.12), &&: EMT signature effect is maintained after adjusting for the T‐cell score (*P* = 0.02). Kaplan–Meier curves for EMT (C, D), Metastasis (E), and their combination (F). Joint effect of T‐cell scores and expression of genes in the DNA methylation metastasis signature (G) and the EMT DNA methylation signatures (H) on melanoma‐specific survival. There was no significant statistical interaction in these analyses. The Melanoma Leeds Cohort contains gene expression profiles for 703 primary melanoma tumors (drug‐naïve). Kaplan–Meier plots and the significance of survival profile differences were computed using the log‐rank test.

In order to evaluate the potential prognostic value of genes regulated by DNA methylation in each signature (Fig. 2), we evaluated gene expression values of 703 primary melanoma samples (drug‐naïve patients when the tissue was sampled in the period 2000–2012) from the Leeds Melanoma Cohort. We averaged gene expression levels from the genes comprising each signature into a single score and evaluated the correlation of high/low scoring samples with melanoma‐specific patient survival (MSS) (Fig. 3A), Breslow thickness (the most important prognostic factor for melanoma) [36], and tumor immune cell scores (Fig. 3B). EMT signature genes were significantly associated with longer MSS (HR = 0.59, *P* = 0.0002) while genes present in the metastasis signature correlated with shorter MSS (HR = 1.49, *P* = 0.005) (Fig. 3A). These findings are also highlighted using Kaplan–Meier plots (Fig. 3C–E).

Consistently with this observation, the EMT expression signature correlated negatively with Breslow thickness and positively with T cell, B cell, and cytotoxic cell counts (immune cell scores) (Fig. 3B). Interestingly, we observed the opposite trend for the combined score of the metastasis signature, in which the expression of these genes was positively correlated with Breslow thickness and negatively correlated with immune cell scores. These results highlight the possible negative correlation between immune infiltrates in tumor and tumor thickness based on epigenetic signatures identified by our murine model of melanoma progression.

We further analyzed jointly these opposing effects on MSS of EMT and metastasis signatures and immune cell scores (Fig. 3F–H). Patients with high EMT signature and low Metastasis signature gene expression scores had the longest survival, and the reverse was true (shortest survival for those with low EMT in conjunction with high metastasis score), reflecting the independence between the two signatures (Fig. 3F). The prognostic effect of the EMT and metastasis signatures was also independent of that of the T‐cell score (Fig. 3G,H). Overall, these results demonstrate that combined analyses of EMT and metastasis gene methylation signatures identified in our model are possible independent prognostic biomarkers for melanoma patients.

### 3.4 Identification of epigenetic driver genes of murine melanoma progression

**Fig. 4:**
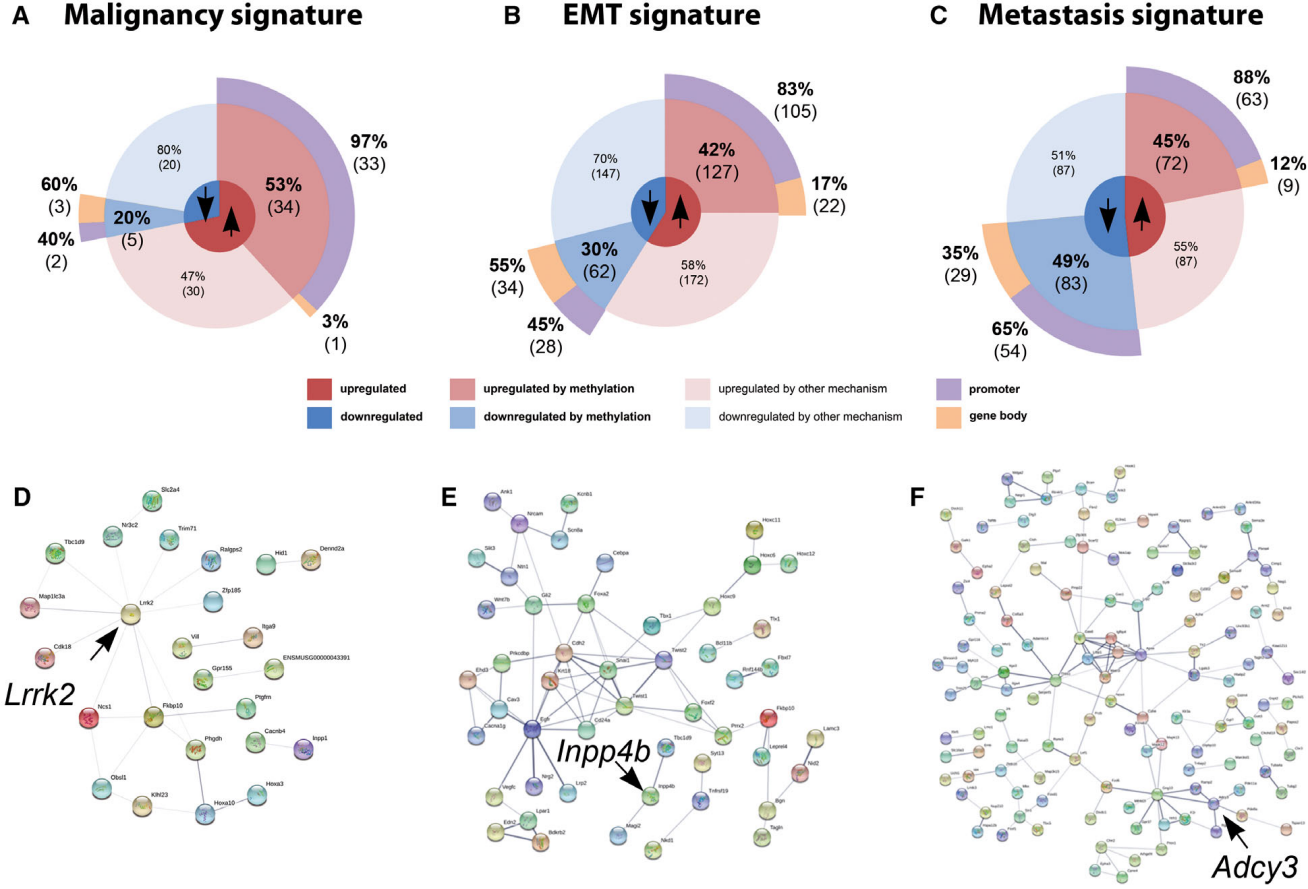
*Lrrk2*, *Inpp4b,* and *Adcy3* are important epigenetically regulated hubs in malignancy, EMT, and metastasis signatures, respectively. Pie charts represent the percentages of genes regulated by DNA methylation in the malignancy (A), EMT (B), and metastasis (C) signatures. (D–F) Protein–protein interaction network (PPI) of genes regulated by DNA methylation in each signature. Genes selected for further studies are indicated by the arrows.

After observing the prognostic value of the methylation signatures identified in our model, we next analyzed each signature to identify epigenetic driver genes. Most of the upregulated genes in each signature with evidence of being epigenetically regulated displayed differential promoter DNA methylation between cell lines (97% in the Malignancy, 83% in the EMT, and 88% in the Metastasis signature), while only a small number of genes regulated by gene body DNA methylation (3% in the malignancy, 17% in the EMT, and 12% in the metastasis signature). Conversely, downregulated genes with evidence of epigenetic regulation are driven by differential promoter (40% in the malignancy, 45% in the EMT, and 65% in the metastasis signature) or gene body (60% in the malignancy, 55% in the EMT, and 35% in the metastasis signature) DNA methylation without significant bias (Fig. 4A–C). These results also demonstrate the well‐known hallmark that cancer‐specific promoter and gene body DNA methylation negatively and positively correlates with gene expression, respectively [4].

In order to search for specific driver genes regulated by DNA methylation in each signature, we analyzed *in silico* protein–protein interactions (PPI) to select important expression hubs that may play a role in melanoma progression and metastasis using the web tool STRING (https://string‐db.org/cgi/network.pl) [37]. We observed weak interactions between malignancy signature proteins (Fig. 4D); however, we identified LRRK2 (leucine‐rich repeat kinase 2) as an interacting partner with most proteins in the signature, being LRRK2 itself upregulated by promoter DNA hypomethylation. LRRK2 is known to interact with proteins in the cellular environment, including proteins involved in MAPK signaling pathway [38, 39], a pathway frequently disrupted in melanoma [40].

We observed strong interactions between proteins in the EMT signature (Fig. 4E). Among those, we identified the *Inpp4b* (inositol polyphosphate 4phosphatase type II) gene, that is downregulated by gene body DNA hypomethylation in the EMT signature, as a potential epigenetic driver. *Inpp4b* was described as a melanoma tumor suppressor via AKT regulation [41] and an oncogene based on its role in activating SGK3 [42].

The PPI of the metastasis signature displayed a large number of proteins and interactions (Fig. 4F). Among these, we identified *Adcy3* (adenylate cyclase 3) as a potential epigenetically regulated gene, as *Adcy3* is upregulated by promoter DNA hypomethylation in this signature. Although this gene has not yet been described in melanoma, it has been described as an oncogene by activating CREB and is overexpressed by promoter DNA hypomethylation in gastric cancer [43].

**Fig. 5:**
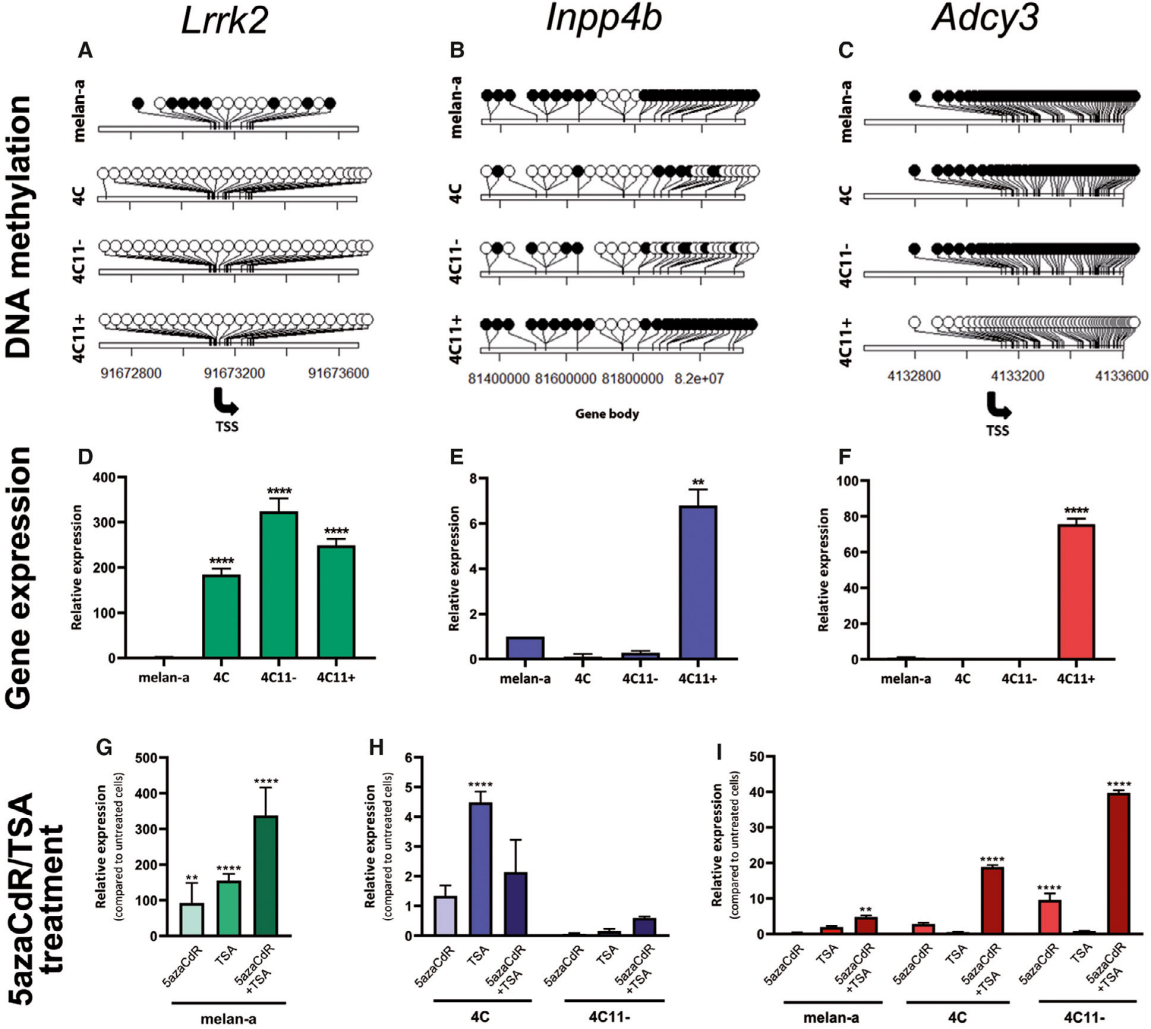
*Lrrk2*, *Inpp4b,* and *Adcy3* are epigenetically regulated. Lolliplots representing the DNA methylation status for each CpG (black: methylated, white: unmethylated, TSS: transcription start site) within selected regions of promoter (*Lrrk2* and *Adcy3*) or gene body (*Inpp4b*) regions for each cell line (A–C). Bar plots illustrating expression values of genes analyzed by RT‐qPCR in each cell line (D–F). Bar plots of expression values in specific cell lines after epigenetic drug treatment (5azaCdR, TSA, and 5azaCdR + TSA) (G–I). Data are represented as mean ± SEM. All experiments were performed in triplicates for each cell line and data are represented as mean ± SEM. Difference between cell lines was analyzed via ANOVA, and adjusted *P*‐values are shown as ****< 0.0001; ***0.0008 < value < 0.0002; **0.007 < value < 0.001; *< 0.05. melan‐a: parental nontumorigenic melanocytes; 4C: premalignant undifferentiated melanocytes; 4C11−: nonmetastatic undifferentiated melanoma cells; 4C11+: metastatic differentiated melanoma cells.

In order to better understand the epigenetic regulation of *Lrrk2*, *Inpp4b,* and *Adcy3* in our murine melanoma model, we analyzed the ERBBS and expression data for CpG resolution methylation levels (Fig. 5A–C). *Lrrk2* displays promoter DNA methylation‐based silencing in melan‐a cells but is completely hypomethylated and upregulated in 4C, 4C11−, and 4C11+ cells (Fig. 5A,D). *Inpp4b* presented substantial gene body DNA methylation in melan‐a and 4C11+ cells but not in 4C and 4C11− cells. These correlated with lower *Inpp4b* expression in 4C and 4C11− cells compared to melan‐a and 4C11+ cells (Fig. 5B,E). Finally, we detected promoter *Adcy3* DNA methylation in melan‐a, 4C and 4C11‐ cells that correlated with gene silencing. However, 4C11+ cells displayed gene activation by promoter DNA hypomethylation (Fig. 5C,F).

We next treated each cell line with the DNA methyltransferase (DNMT) inhibitor 5‐Aza‐2’‐deoxycytidine (5azaCdR) and/or the histone deacetylase inhibitor Trichostatin A (TSA) to determine how promoter or gene body DNA methylation drives expression of *Lrrk2*, *Inpp4b,* and *Adcy3*. *Lrrk2* was upregulated in melan‐a cells after treatment of 5azaCdR and/or TSA (Fig. 5G), *Inpp4b* expression did not significantly change after treatment of 4C and 4C11‐ cells with 5azaCdR alone (Fig. 5H); however, the reactivation of *Inpp4b* in 4C cells after the treatment with TSA, a specific inhibitor of HDAC class I/II, suggests that chromatin modifications may be important in regulating expression of this gene in premalignant melanocytes. Interestingly, neither 5azaCdR nor TSA substantially increased *Inpp4b* expression in 4C11‐ cells, indicating that other mechanisms might regulate expression of this gene in nonmetastatic melanoma cells. Lower *Adcy3* promoter DNA methylation was observed in 4C11+ cells when compared to the other cell lines, and as expected, 4C11+ cells showed higher *Adcy3* gene expression (Fig. 5F). Finally, we observed a significant increase in *Adcy3* gene expression in melan‐a, 4C, and 4C11− cell lines after combined treatments of 5azaCdR and TSA (Fig. 5I), suggesting that DNA demethylation is required for *Adcy3* reactivation.

### 3.5 *Adcy3* and *Inpp4b* are potential oncogenes in melanoma

**Fig. 6:**
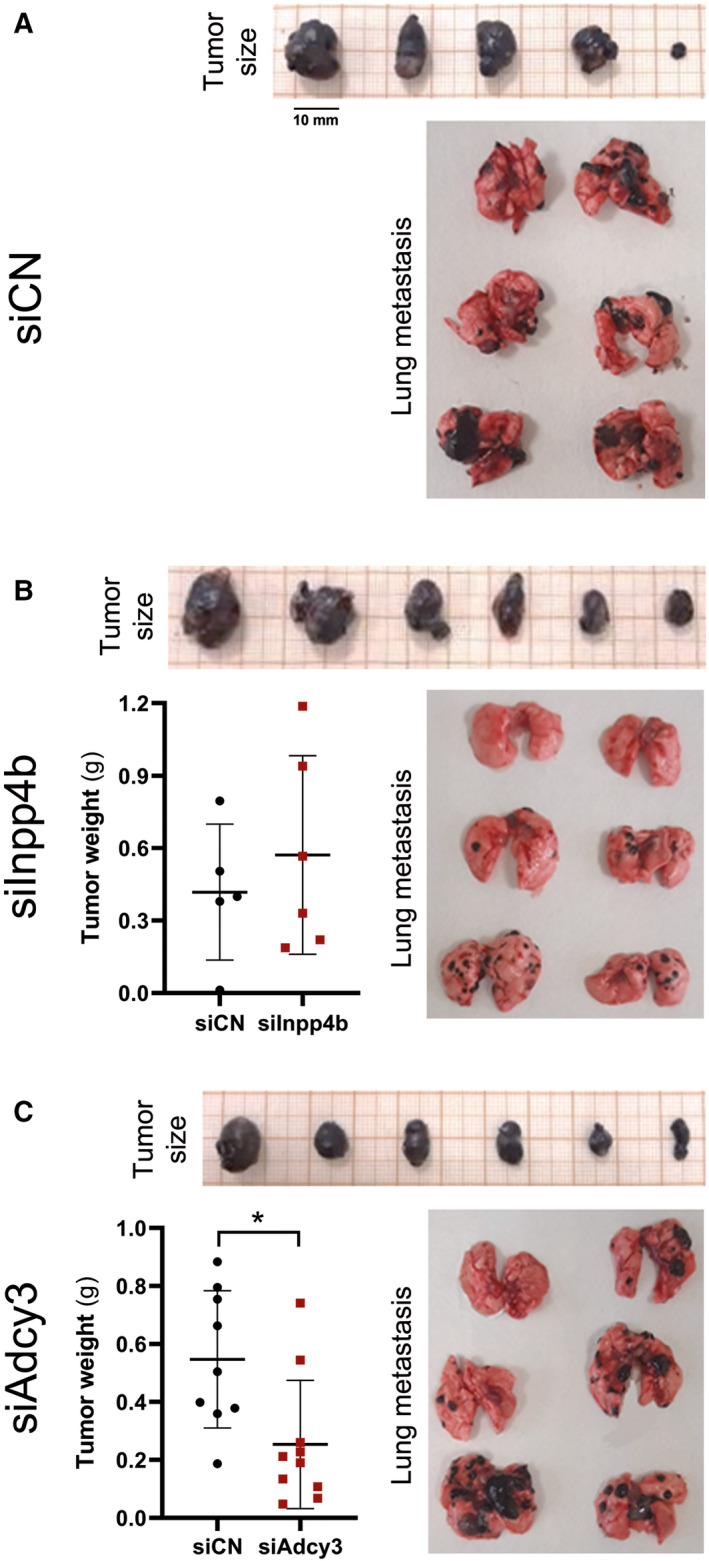
*Inpp4b* and *Adcy3* promote *in vivo* metastasis formation and tumor growth, respectively. Metastatic 4C11+ cells were transfected with a control siRNA (siCN) (A), siRNA directed to *Inpp4b* (siInpp4b) (B) and *Adcy3* (siAdcy3) (C). Dot plots illustrating differences between tumor weight of animals injected siAdcy3, siInpp4b, or siCN. The experiments were performed in triplicates with six animals per group. Data are represented as mean ± SEM, and difference between groups was analyzed via ANOVA, and adjusted *P*‐values are shown as ****< 0.0001; ***0.0008 < value < 0.0002; **0.007 < value < 0.001; *< 0.05.

We next determined how *Adcy3* and *Inpp4b in vivo* tumor growth and *in vivo* metastatic potential using siRNA knockdown technology. We selected the 4C11+ cell line for this analysis, as this cell line displays the highest expression of each of these genes and is the most aggressive cell line in the linear model. *In vivo* tumor growth was determined by subcutaneously injecting *Adcy3‐* or *Inpp4b*‐silenced 4C11+ cells into mice flank region. Mice injected with *Adcy3*‐silenced 4C11+ cells (siAdcy3) developed smaller tumors in size and weight when compared to controls (Fig. 6A,B), suggesting that this gene may play an important role in tumor growth and development. *Inpp4b*‐silenced 4C11+ cells (siInpp4b) did not result in any significant differences in tumor size and weight when compared to control cells (Fig. 6A,C).

We evaluated metastatic formation in mouse lungs, a common site for melanoma metastases, by intravenously injecting 4C11+ cells silenced for *Adcy3*, *Inpp4b,* or control cells. For *Adcy3‐*silenced cells, we did not observe any difference regarding metastatic foci size and cell number; however, we noticed a clear decrease in the size of metastatic foci in mice injected with *Inpp4b*‐silenced cells, suggesting a role for *Inpp4b* in promoting metastasis (Fig. 6A–C). Together, these results show for the first time the potential role of *Adcy3* in *in vivo* tumor growth and the involvement of *Inpp4b* in tumor aggressiveness and metastasis formation.

### 3.6 *Lrrk2*, *Adcy3*, and *Inpp4b* DNA methylation levels have prognostic value for melanoma patients

We analyzed *LRRK2*, *ADCY3*, and *INPP4B* DNA methylation and expression profiles in human melanoma cohorts to determine whether the murine model data are applicable to human melanoma biology. Using the Melanoma Explorer web tool [26], we analyzed publicly available DNA methylation, gene expression, and patient outcome data from The Cancer Genome Atlas (TCGA), the Sweden Melanoma Cohort (GSE51547), and the Bogunovic Cohort (GSE19234) (Table S1).

**Fig. 7:**
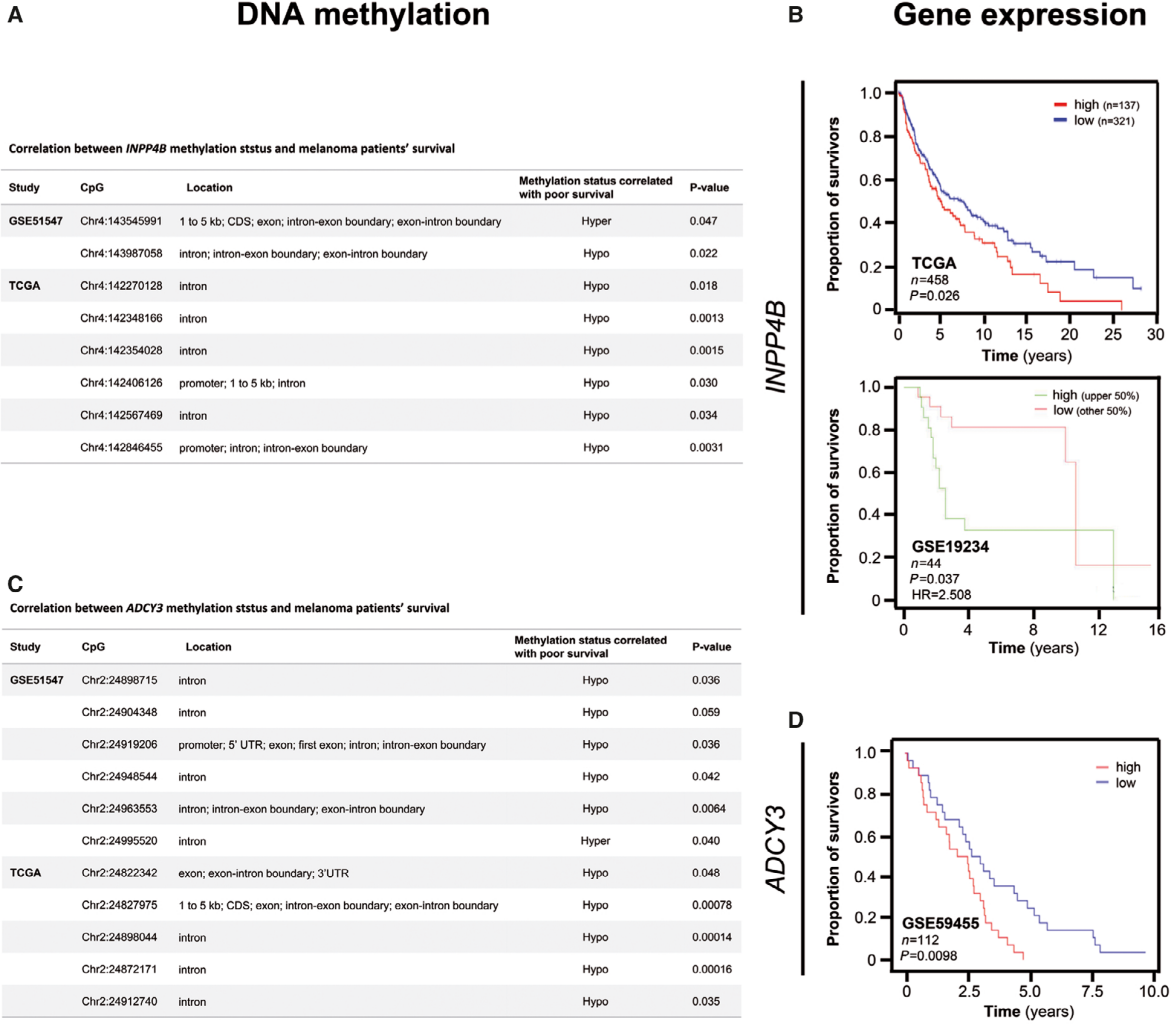
DNA methylation as prognostic marker for melanoma patient survival. Specific melanoma patient cohorts, CpG genomic coordinates, genomic locations (GRCh38 annotation), survival, and DNA methylation status for *INPP4B* (A) and *ADCY3* (C). Kaplan–Meier curves in years for gene expression values of patients from cohorts identified in the lower‐left corner, followed by number of patients (*n*), *P*‐value and hazard ratio (HR) for *INPP4B* (B) and *ADCY3* (D). Number of patients (*n*) for each cohort is shown inside Kaplan–Meier plots.

*LRRK2* DNA methylation at gene body and promoter region did not correlate with patient survival based on TCGA data; however, we noticed that higher *LRRK2* expression significantly correlated with improved patient survival in this same cohort (Fig. S2). DNA hypomethylation of several CpG sites within the *INPP4B* locus was significantly correlated with poor survival (TCGA and Swedish cohorts); however, DNA hypermethylation of one CpG site correlated with poor survival (Swedish cohort) (Fig. 7A). The majority of CpGs were located within an intron or intron–exon boundary; this is similar to our murine data in which differential *Inpp4b* DNA methylation was identified in its gene body regions.

Gene expression data of primary melanomas from both TCGA and Bogunovic Cohorts [44] showed that higher *INPP4B* gene expression values correlated with poor patient survival (Fig. 7B), underscoring the prognostic importance of *INPP4B* DNA methylation and gene expression in melanoma patients.

The DNA methylation levels of 11 CpGs located in *ADCY3* were significantly correlated with poor survival in GSE51547 and TCGA cohorts, in which 10 CpGs displayed DNA hypomethylation and one CpG displayed DNA hypermethylation (Fig. 7C). Increased *ADCY3* gene expression from the Hunter Australians Cohort (GSE59455) [45] also correlated with poor survival (Fig. 7D). These findings are consistent with results generated with our murine model, in which the most aggressive cell lines exhibit high gene expression driven by promoter DNA hypomethylation or gene body DNA hypermethylation.

## 4 Discussion

Epigenetic alterations, such as DNA methylation, can influence gene expression and has promise for cancer patient stratification, prognosis, and precise therapeutic intervention [8, 46, 47]. In this study, we analyzed changes in gene expression that are regulated by promoter or gene body DNA methylation using a clinically relevant mouse melanoma model, in which tumorigenesis is triggered by continuous stress conditions leading to epigenetic alterations rather than specific mutations. We have previously characterized the transcriptome profiles of the cell lines comprising this linear model and identified sets of differentially expressed genes (DEGs) at each transition step of tumor progression, and transcriptional signatures related to malignancy, metastasis, and epithelial‐to‐mesenchymal transition [13]. The integrated analysis of DNA methylation and gene expression data reported in the present study shows that the transcriptional changes during melanoma progression are largely driven by promoter DNA hypermethylation, as well as global genome DNA hypomethylation. We also observed the positive correlation between gene body DNA methylation and gene expression which is frequently overlooked but has prognostic and therapeutic potential [3, 48, 49]. Pairwise comparisons between cell lines in the model showed that 40–87% of genes with altered expression are regulated by promoter DNA methylation, while 13–58% of differently expressed are regulated by gene body DNA methylation.

We investigated the prognostic value of genes that are regulated by promoter or gene body DNA methylation and comprise *Malignancy*, *EMT,* and *Metastasis* signatures. The results observed in the Leeds Melanoma Cohort highlight associations of *Metastasis* and *EMT* signatures with melanoma‐specific survival. These observations are consistent with the view that the most aggressive primary melanomas have a higher metastatic potential [50]. On the other hand, primary melanomas with a higher EMT signature expression score show better prognosis and therefore a less aggressive phenotype. This observation emphasizes that melanoma EMT transition process is not as linear as we expect; however, it comprises multiple‐step process and not all of the stages may be as aggressive as expected [51]. Once more, this result highlights the plasticity and heterogeneity within the tumor environment even at early stages of disease [52], as well as the importance of each subtype for improved prognostic and drug targeting purposes. Another interesting finding was that high EMT gene expression and high T‐cell scores in primary tumors were correlated with better prognosis. However, it is usually observed a more aggressive and resistant phenotype in EMT cells [53]. Once more, we can observe the complexity of this process involving different stages of what we call EMT phenotype and tumor stages.

The two phenotypes first described in melanoma are the *invasive*, characterized by slow proliferative rate and low MITF levels; and the *proliferative*, associated with high proliferation and MITF levels [54]. Melanoma cells are also capable of a process called ‘phenotype switching’ between different states that regulate drug resistance and tumor plasticity [55, 56]. Nevertheless, melanoma murine model cell lines also present different phenotypes: 4C and 4C11− cells display an undifferentiated and mesenchymal‐like phenotype, while 4C11+ cells have a pigmented, differentiated, and highly proliferative phenotype, as described previously in different subtypes within melanoma tumor environment [56, 57, 58]. Results of pathway enrichment analysis of epigenetically regulated genes present in malignancy, EMT, and metastasis signatures highlighted the different cell states observed along our model. For instance, genes identified in EMT signature were enriched with developmental processes while metastasis genes were enriched with neural cell processes and a well‐known altered pathway in melanoma (ERK1 and ERK2). These changes may be regulated by microenvironmental signals involving epigenetic alterations [59] that are emphasized by identifying outcomes based on unique signatures in melanoma patients, as well as observed in gene pathway enrichment analysis of genes present in each signature.

We identified two epigenetic driver genes (*INPP4B* and *ADCY3*) that are potential candidates for specific targeted therapies. *In vivo* studies showed *INPP4B* as potentially involved in metastasis formation and therefore related to tumor aggressiveness. *INPP4B* was described as an oncogene by SGK3 activation in melanoma [42], breast cancer [60], and colon cancer [61]. Therefore, its function as an oncogene is emphasized as it increases the metastatic potential of melanoma cells. Nevertheless, additional studies are needed to understand its role in molecular signaling pathways and as a potential drug target.

*ADCY3* is an adenylate cyclase able to catalyze the formation of cAMP, an important second messenger able to activate PKA (protein kinase A) and EPAC (exchange factor directly activated by cAMP) [62, 63]. Although *ADCY3* has not yet been described in melanoma, it has been described as an oncogene in gastric cancer, in which promoter DNA hypomethylation drives its overexpression and CREB activation [43]. In our murine model, *Adcy3* may play a role in tumor formation in metastatic melanoma; however, metastatic potential remained unchanged after *Adcy3* knockdown. Therefore, we hypothesize that the gene may be involved in proliferation rather than metastasis, although additional experiments are required to determine its role in proliferation.

Preclinical research models have been widely used to validate therapeutic drug efficacy and identify targets for precision medicine [64]. Importantly, the epigenetic driver genes identified in this study predict melanoma patient outcome in multiple cohorts, highlighting the potential of the model to identify epigenetic alterations during melanoma progression and metastasis. Therefore, we suggest that our model is important for preclinical melanoma research. Further studies of gene regulation by DNA methylation in melanoma patients will identify biomarkers for prognosis, as well as patient stratification for epigenetic therapies to reverse these alterations.

## 5 Conclusion

Adenylate cyclase type 3 (*Adcy3*) and inositol polyphosphate 4‐phosphatase type II (*Inpp4b*) were identified as genes regulated by DNA methylation that, respectively, affect melanoma growth and metastasis. Importantly, potential prognostic markers, found based on the gene expression and DNA methylation profiles in a murine model of melanoma progression, were validated in a large cohort of primary melanoma patients. These data emphasize the value of our murine model of melanoma progression for preclinical melanoma research.

## Conflict of interest

The authors declare no conflict of interest.

## Author contributions

Conceptualization, DDP, MGJ, and GL; Methodology, DAP, FER, DP, CO, JN, JNB, EMR, CEM, GL, MGJ; Investigation, DAP, FER, APA, DP, HG, JN; Writing—Original Draft, DAP; Visualization, DAP, MGJ; Writing—Review and Editing, MGJ, GL, DJW, CEM, EMR; Funding Acquisition, MGJ; Resources, MGJ, GL, EMR, CEM, JNB; Supervision, MGJ, and GL.

### Peer Review

The peer review history for this article is available at https://publons.com/publon/10.1002/1878‐0261.13185.

## Supporting information


**Fig. S1**. Expression of differentially expressed genes regulated by promoter or gene body DNA methylation.

**Fig. S2**. Correlation between *LRRK2* expression data and melanoma survival.

**Table S1**. Melanoma cohorts and data information.

**Table S2**. List of genes with both gene body and promoter differently methylated.

**Table S3**. List of genes of each signature.

Supplementary Material

## Data Availability

The RNA‐seq data are accessible through the Gene Expression Omnibus (https://www.ncbi.nlm.nih.gov/geo/) under the accession number GSE149884. The ERRBS data generated during this study are available at https://github.com/flaviaerius/methylation‐data/tree/master/input/raw‐methylation‐data.
