# Supplementary material for: Genes regulated by DNA methylation are involved in distinct phenotypes during melanoma progression and are prognostic factors for patients
Source: Mol Oncol. 2022 Feb 4;16(9):1913–30. doi: 10.1002/1878-0261.13185 (PMC9067153; doi:10.1002/1878-0261.13185)
Supplement: Supplementary file 1 — Fig. S1. Expression of differentially expressed genes regulated by promoter or gene body DNA methylation. [file MOL2-16-1913-s003.pdf]

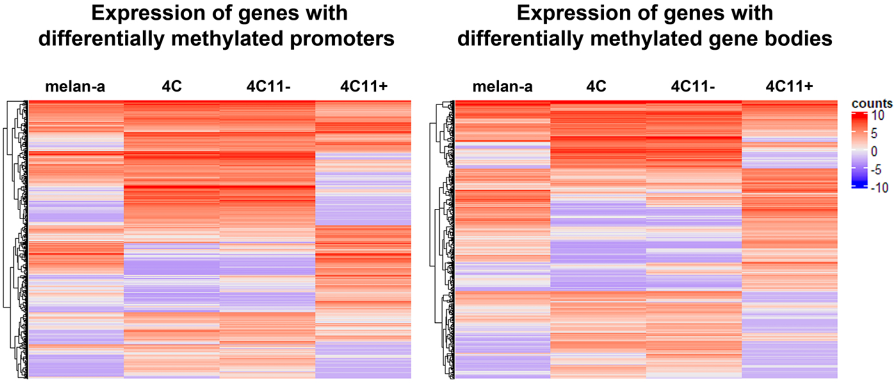

Expression of genes with differentially methylated promoters
melan-a
4C
4C11-
4C11+
Expression of genes with differentially methylated gene bodies
melan-a
4C
4C11-
4C11+
counts
10
5
0
-5
-10
