# Supplementary figures and images for: Genes regulated by DNA methylation are involved in distinct phenotypes during melanoma progression and are prognostic factors for patients
Source: Mol Oncol. 2022 Feb 4;16(9):1913–30. doi: 10.1002/1878-0261.13185 (PMC9067153; doi:10.1002/1878-0261.13185)

LRRK2

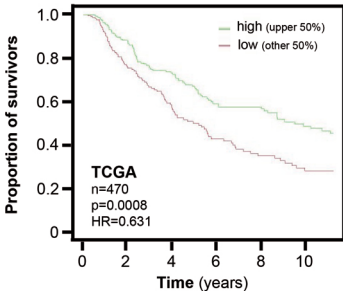

Supplement: Supplementary file 2 — Fig. S2. Correlation between LRRK2 expression data and melanoma survival. [file MOL2-16-1913-s005.pdf]
