# Supplementary material for: Genes regulated by DNA methylation are involved in distinct phenotypes during melanoma progression and are prognostic factors for patients
Source: Mol Oncol. 2022 Feb 4;16(9):1913–30. doi: 10.1002/1878-0261.13185 (PMC9067153; doi:10.1002/1878-0261.13185)
Supplement: Supplementary file 3 — Table S1. Melanoma cohorts and data information. [file MOL2-16-1913-s002.pdf]

Data set name	Analysis	Repository ID	# Samples	Methodology	Reference	Tool
**Leeds melanoma cohort**	mRNA	EGAS00001002922	**703** primary melanomas (drug-naïve)	Illumina DASL Human HT12 v4 array	Nsengimana et al., 2018; Muralidhar et al., 2019	-
**TCGA SKCM cohort**	mRNA	G.D.C. v.4.0	**468** primary and metastatic melanomas	Illumina HiSeq 2000	Akbani et al., 2015	Melanoma Explorer[1]; Oncolnc[2]; Precog[3]
	DNA methylation	G.D.C. v.4.0	**470** primary and metastatic melanomas	The Illumina Infinium HM450 array	Akbani et al., 2015	Melanoma Explorer[1]
	Protein	G.D.C. v.4.0	**354** primary and metastatic melanomas	RPPA	Akbani et al., 2015	TRGAted[4]
**Sweden melanoma samples**	mRNA	GSE65904	**214** primary and metastatic melanomas	Illumina HumanHT-12 version 4 microarray	Cirenajwis et al., 2015	Melanoma Explorer[1]
	DNA methylation	GSE51547	**50** metastatic melanomas	Illumina HumanMethylation450 microarray	Lauss et al., 2015	Melanoma Explorer[1]
**Hunter Australians cohort**	mRNA	GSE59455	**141** primary and metastatic melanomas	Illumina HumanRef-8 WG-DASL v3.0 microarray	Budden et al., 2016	Melanoma Explorer[1]
**Talantov cohort**	mRNA	GSE3189	**50** normal skin, benign nevi and primary melanomas	Affymetrix Hu133A microarray	Talantov et al., 2005	Oncomine[5]
**Xu cohort**	mRNA	GSE8401	**83** primary and metastatic melanomas	Affymetrix Human Genome U133A Array	Xu et al., 2008	Oncomine[5]
**Bogunovic cohort**	mRNA	GSE19234	**44** metastatic melanomas	Affymetrix Human Genome U133 Plus 2.0 Array	Bogunovic et al., 2009	Oncomine[5]; OSskcm[6]

References

Akbani et al (The Cancer Genome Atlas Network). Genomic Classification of Cutaneous Melanoma. *Cell* **161**: 1681–1696 (2015)

Bogunovic D, O'Neill DW, Belitskaya-Levy I, Vacic V, Yu YL, Adams S, Darvishian F, Berman R, Shapiro R, Pavlick AC, Lonardi S, Zavadil J, Osman I, Bhardwaj N. Immune profile and mitotic index of metastatic melanoma lesions enhance clinical staging in predicting patient survival. *Proc. Natl. Acad. Sci. U.S.A.* **106**(48): 20429-20434 (2009)

Budden T, Davey RJ, Vilain RE, Ashton KA, Braye SG, Beveridge NJ, Bowden NA. Repair of UVB-induced DNA damage is reduced in melanoma due to low XPC and global genome repair. *Oncotarget* **7**(38): 60940-60953 (2016)

Cirenajwis H, Ekedahl H, Lauss M, Harbst K, Carneiro A, Enoksson J, Rosengren F, Werner-Hartman L, Törngren T, Kvist A, Fredlund E, Bendahl PO, Jirström K, Lundgren L, Howlin J, Borg Å, Gruvberger-Saal SK, Saal LH, Nielsen K, Ringnér M, Tsao H, Olsson H, Ingvar C, Staaf J, Jönsson G. Molecular stratification of metastatic melanoma using gene expression profiling: Prediction of survival outcome and benefit from molecular targeted therapy. *Oncotarget* **6**(14): 12297-12309 (2015)

Lauss M, Ringnér M, Karlsson A, Harbst K, Busch C, Geisler J, Lønning PE, Staaf J, Jönsson G. DNA methylation subgroups in melanoma are associated with proliferative and immunological processes. *BMC Med. Genomics* **8**: 73 (2015)

Muralidhar S, Filia A, Nsengimana J, Poźniak J, O'Shea SJ, Diaz JM, Harland M, Randerson-Moor JA, Reichrath J, Laye JP, van der Weyden L, Adams DJ, Bishop DT, Newton-Bishop J. Vitamin D-VDR Signaling Inhibits Wnt/β-Catenin-Mediated Melanoma Progression and Promotes Antitumor Immunity. *Cancer Res*. **79**(23): 5986-5998 (2019)

Nsengimana J, Laye J, Filia A, O'Shea S, Muralidhar S, Poźniak J, Droop A, Chan M, Walker C, Parkinson L, Gascoyne J, Mell T, Polso M, Jewell R, Randerson-Moor J, Cook GP, Bishop DT, Newton-Bishop J. β-Catenin-mediated immune evasion pathway frequently operates in primary cutaneous melanomas. *J. Clin. Invest*. **128**(5): 2048-2063 (2018)

Talantov D, Mazumder A, Yu JX, Briggs T, Jiang Y, Backus J, Atkins D, Wang Y. Novel genes associated with malignant melanoma but not benign melanocytic lesions. *Clin. Cancer Res*. **11**(20): 7234-7242 (2005)

Xu L, Shen SS, Hoshida Y, Subramanian A, Ross K, Brunet JP, Wagner SN, Ramaswamy S, Mesirov JP, Hynes RO. Gene expression changes in an animal melanoma model correlate with aggressiveness of human melanoma metastases. *Mol. Cancer Res*. **6**(5): 760-769 (2008)

References (Tools)

[1] Strbenac D, Wang K, Wang X, Dong J, Mann GJ, Mueller S, Yang JYH. Melanoma Explorer: a web application to allow easy reanalysis of publicly available and clinically annotated melanoma omics data sets. *Melanoma Res*. **29**(3): 342-344 (2019)

[2] Anaya J. OncoLnc: linking TCGA survival data to mRNAs, miRNAs, and lncRNAs. *PeerJ Computer Science* **2**: e67 (2016)

[3] Gentles A, Newman A, Liu C, Bratman SV, Feng W, Kim D, Nair VS, Xu Y, Khuong A, Hoang CD, Diehn M, West RB, Plevritis SK, Alizadeh AA. The prognostic landscape of genes and infiltrating immune cells across human cancers. *Nat. Med.* **21**: 938–945 (2015)

[4] Borcherding, N., Bormann, N., Voigt, A., & Zhang, W. TRGAted: A web tool for survival analysis using protein data in the Cancer Genome Atlas. *F1000Research* **7**: 1235 (2018)

[5] Rhodes DR, Yu J, Shanker K, Deshpande N, Varambally R, Ghosh D, Barrette T, Pandey A, Chinnaiyan AM. ONCOMINE: a cancer microarray database and integrated data-mining platform. *Neoplasia* **6**(1): 1-6 (2004)

[6] Zhang L, Wang Q, Wang L, Xie L, An Y, Zhang G, Zhu W, Li Y, Liu Z, Zhang X, Tang P, Huo X, Guo X. OSskcm: an online survival analysis webserver for skin cutaneous melanoma based on 1085 transcriptomic profiles. *Cancer Cell International* **20**(1) (2020)
