# Supplementary material for: Genes regulated by DNA methylation are involved in distinct phenotypes during melanoma progression and are prognostic factors for patients
Source: Mol Oncol. 2022 Feb 4;16(9):1913–30. doi: 10.1002/1878-0261.13185 (PMC9067153; doi:10.1002/1878-0261.13185)
Supplement: Supplementary file 6 — Supplementary Material [file MOL2-16-1913-s006.docx]

**SUPPLEMENTARY INFORMATION**

**LEGENDS TO SUPPLEMENTARY FIGURES AND TABLES**

**Figure S1. Expression of differentially expressed genes regulated by promoter or gene body DNA methylation.** Heatmaps showing the expression (log Fold Change (logFC) of genes based on pairwise comparisons between cell lines stratified by promoter or gene body DNA methylation alterations. melan-a: parental nontumorigenic melanocytes; 4C: pre-malignant undifferentiated melanocytes; 4C11-: non-metastatic undifferentiated melanoma cells; 4C11+: metastatic differentiated melanoma cells.

**Figure S2. Correlation between *LRRK2* expression data and melanoma survival.** Kaplan-Meier curve in years for gene expression values (above and below the median) of melanoma patients from the TCGA cohort, followed by number of patients (n), p-value and hazard ratio (HR) for *LRRK2* gene*.*

**Table S1. Melanoma cohorts and data information.** Information about each melanoma cohort, number of samples, methodologies, references and tools used in the study.

**Table S2. List of genes with both gene body and promoter differently methylated.** Table with Ensembl gene ID, Gene symbol, comparison between cell lines and gene expression status.

**Table S3. List of genes of each signature.** Table with Ensembl gene ID, Gene symbol, gene DNA methylation status of the gene, region of DNA methylation, gene expression status (second cell line compared to the first one) and the identified signature.
